# Synergistic effects of metformin with liraglutide against endothelial dysfunction through GLP-1 receptor and PKA signalling pathway

**DOI:** 10.1038/srep41085

**Published:** 2017-02-01

**Authors:** Jing Ke, Ye Liu, Jin Yang, Ran Lu, Qing Tian, Wenfang Hou, Guang Wang, Rui Wei, Tianpei Hong

**Affiliations:** 1 Department of Endocrinology and Metabolism, Peking University Third Hospital, Beijing, China; 2 Department of Endocrinology and Metabolism, Beijing Chaoyang Hospital, Capital Medical University, Beijing, China

## Abstract

Metformin or glucagon-like peptide-1 (GLP-1) analogue liraglutide has cardiovascular benefits. However, it is not clear whether their combined treatment have additive or synergistic effects on the vasculature. In this study, human umbilical vein endothelial cells (HUVECs), exposed to palmitic acid (PA) to induce endothelial dysfunction, were incubated with metformin, liraglutide or their combination. High fat diet (HFD)-fed ApoE^−/−^ mice were randomized into control, metformin, liraglutide, and combination treatment groups. Results showed that in PA-treated HUVECs and HFD-fed ApoE^−/−^ mice, combination of metformin and liraglutide at lower dose significantly improved endothelial dysfunction compared with the single treatment. Metformin upregulated GLP-1 receptor (GLP-1R) level and protein kinase A (PKA) phosphorylation. However, PKA inhibition but not GLP-1R blockade eliminated the protective effects of metformin on endothelial function. Furthermore, AMPK inhibitor compound C abolished the metformin-mediated upregulation of GLP-1R level and PKA phosphorylation. In conclusion, combination of metformin and liraglutide has synergistic protective effects on endothelial function. Moreover, metformin stimulates GLP-1R and PKA signalling via AMPK-dependent pathway, which may account for its synergistic protective effects with liraglutide. Our findings provide new insights on the interaction between metformin and GLP-1, and provide important information for designing new GLP-1-based therapy strategies in treating type 2 diabetes.

Cohort studies and meta-analyses indicate that type 2 diabetes is strongly associated with an increased risk of many atherosclerotic cardiovascular diseases^1^,^2^. Although the precise mechanisms responsible for the accelerated atherosclerosis are not fully understood, it has been shown that endothelial dysfunction is one of the earliest events in the development of atherosclerosis. Endothelial dysfunction is characterized by reduced nitric oxide (NO) synthesis and/or bioavailability within peri-endothelial environment, where NO is responsible for maintenance of vascular tissue integrity^3^.

Metformin is the most widely prescribed drug to treat hyperglycaemia in patients with type 2 diabetes. Clinical trials have shown the beneficial effects of metformin on cardiovascular system^4^,^5^,^6^. Moreover, metformin can directly protect endothelial cells from injury, which is beyond its glucose-lowering effects^7^,^8^. Glucagon-like peptide-1 (GLP-1)-based agents (including GLP-1 analogues and dipeptidyl peptidase 4 inhibitors) have been recently approved as new therapeutic options for patients with type 2 diabetes, based upon the evidence that GLP-1 can stimulate glucose-dependent insulin secretion from pancreatic β cells. Besides its glucose-lowering effect, GLP-1 has other extra-pancreatic effects. Recently, LEADER (Liraglutide Effect and Action in Diabetes: Evaluation of cardiovascular outcome Results) study shows that GLP-1 analogue liraglutide treatment reduces the rates of cardiovascular events and death in patients with type 2 diabetes^9^. Importantly, vascular endothelium is known to play an important role in the maintenance of cardiovascular function^10^. Emerging evidence indicates that GLP-1 and its analogues have direct protective effects on vascular endothelium^11^.

In a clinical trial, combination therapy with liraglutide and metformin shows significantly greater weight loss and lower incidence of hypoglycaemia, as compared to combination therapy with glimepiride and metformin^12^,^13^. Moreover, it has been reported that in type 2 diabetic patients with adequate glycaemic control by metformin monotherapy, several markers of cardiovascular risk are improved by addition of liraglutide treatment, suggesting that metformin and liraglutide may have additive or even synergistic effects on vasculature^14^. However, whether this combination therapy has a synergistic effect on endothelial function remains unclear, due to lack of related experimental evidence. In this study, therefore, we aimed to investigate the effects of combination treatment with metformin and liraglutide on lipotoxicity-induced endothelial dysfunction and to explore the underlying mechanism.

## Results

### Combination treatment with metformin and liraglutide is more effective than single treatment to protect against palmitic acid (PA)-induced endothelial dysfunction

**Figure 1:**
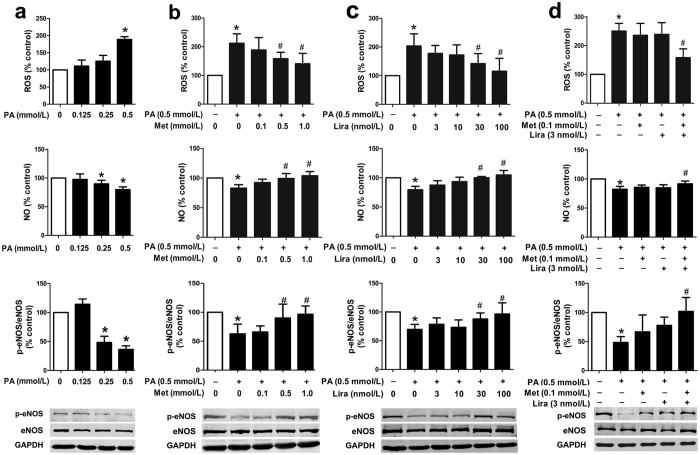
Synergistic effects of metformin and liraglutide on PA-induced endothelial dysfunction in primary HUVECs. (**a**) Dose-dependent effects of PA on endothelial function in HUVECs, including intracellular ROS production (upper panel), supernatant NO level (middle panel), and p-eNOS protein level (lower panel). HUVECs were pre-treated with different concentrations of metformin (**b**) and liraglutide (**c**) alone or the combination of both (**d**) for 2 h and then exposed to PA for additional 22 h. Data are shown as means ± SD. n = 4. *P < 0.05 (vs. control); ^#^*P* < 0.05 (vs. PA). Met, metformin; Lira, liraglutide.

In cultured human umbilical vein endothelial cells (HUVECs), PA induced endothelial dysfunction, characterized by increased reactive oxygen species (ROS) production, decreased NO level and reduced intracellular phosphorylated endothelial NO synthase (p-eNOS) protein level, in a concentration-dependent manner (Fig. 1a). PA (0.5 mmol/L)-induced endothelial dysfunction was attenuated by single treatment with higher dose of metformin (0.5 and 1.0 mmol/L) or liraglutide (30 and 100 nmol/L). However, there was no such protective effect at lower dose of metformin (0.1 mmol/L) or liraglutide (3 and 10 nmol/L) (Fig. 1b,c). Notably, combination treatment with lower dose of metformin (0.1 mmol/L) and liraglutide (3 nmol/L) had a significant protective effect on the PA-induced upregulation of ROS production and reduction of NO level and p-eNOS protein level (Fig. 1d, Supplemental Fig. 1), suggesting that combination treatment was more effective than single treatment to protect against PA-induced endothelial dysfunction.

### Effects of combination therapy with metformin and liraglutide on vascular endothelial function in ApoE^−/−^ mice

**Figure 2:**
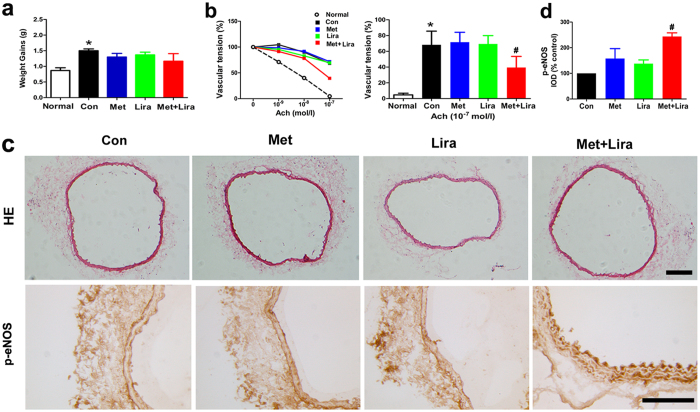
The effects of combined treatment with metformin and liraglutide on vascular function in ApoE^−/−^ mice. Eight-week-old male ApoE^−/−^ mice were fed with high fat diet (HFD) for eight weeks and treated with metformin (100 mg/kg per day, via drinking water) and/or liraglutide (30 μg/kg per day, ip, twice daily) during the final four weeks. (**a**) Weight gains; (**b**) Vascular function as indicated by acetylcholine (Ach)-mediated endothelium-dependent vasodilation in aortic rings; (**c**) Representative images of HE staining and p-eNOS immunostaining; (**d**) Quantitation of immunostaining in the vascular tissue. Normal denotes wild-type mice fed with normal diet, while Con (control), Met (metformin) and Lira (liraglutide) denote HFD-fed ApoE^−/−^ mice without or with the corresponding treatment. Data are shown as means ± SD. n = 6. *P < 0.05 (vs. normal); #*P*  < 0.05 (vs. control). Scale bars: 100 μm.

To verify vascular protective effects of the combination therapy *in vivo*, we used high fat diet (HFD)-fed ApoE^−/−^ mice as our model. After four weeks of treatment with metformin or liraglutide alone or in combination, there was no obvious change of body weight in each group (Fig. 2a). In our experiment of endothelium-dependent vasodilation, 10^−9^–10^−7^ mol/L acetylcholine (Ach) stimulation decreased the vascular tension of aortic rings in a dose-dependent manner in wild-type mice under normal diet condition (Fig. 2b). In HFD-fed ApoE^−/−^ mice, however, Ach (10^−7^ mol/L) stimulation decreased the vascular tension to 68% in control group, 72% in metformin group and 69% in liraglutide group respectively, suggesting that monotherapy with either metformin or liraglutide did not improve vascular reactivity obviously. By contrast, the vascular tension was decreased to 39% in response to 10^−7^ mol/L Ach stimulation in the combination group, which was much better than that in the monotherapy and control groups (Fig. 2b). In line with this observation, p-eNOS protein levels in aortas were upregulated in the combination group compared with the monotherapy and control groups (Fig. 2c,d), although there was no obvious change of vascular endothelial structure in the aortic tissues of each group as indicated by haematoxylin and eosin (HE) staining (Fig. 2c).

### Metformin-induced upregulation of GLP-1 receptor (GLP-1R) level and protein kinase A (PKA) phosphorylation may contribute to the synergistic protective effects of the combination therapy

**Figure 3:**
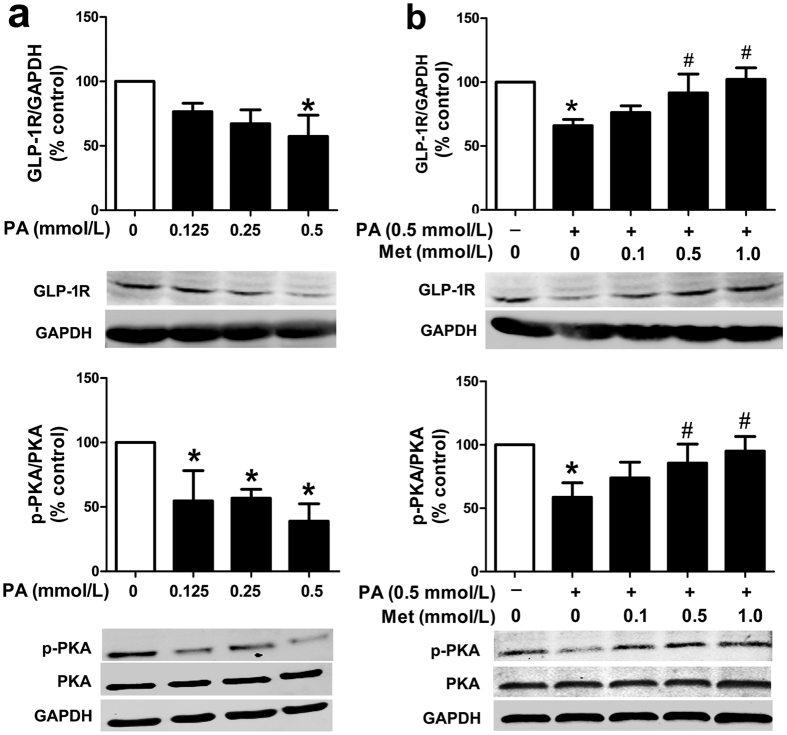
Metformin restored PA-impaired GLP-1R and PKA signalling in HUVECs. (**a**) Impaired GLP-1R level and PKA phosphorylation in PA-treated HUVECs. (**b**) Dose-dependent effects of metformin on GLP-1R level and PKA phosphorylation in PA-impaired HUVECs. The protein levels of GLP-1R or p-PKA were normalized to GAPDH or total PKA proteins, respectively. Data are shown as means ± SD. n = 4. *P < 0.05 (vs. control); ^#^*P* < 0.05 (vs. PA). Met, metformin.

**Figure 4:**
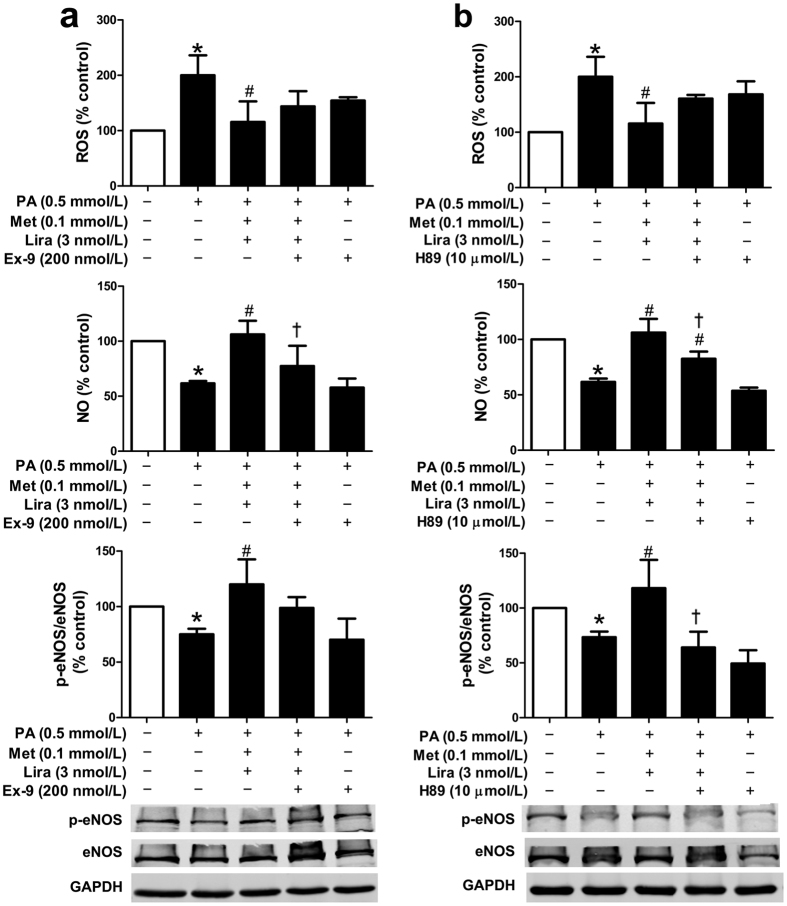
GLP-1R and PKA signalling pathway were involved in the synergistic protective effects of metformin and liraglutide in HUVECs. Cells were incubated with GLP-1R antagonist exendin (9–39) (**a**) or PKA inhibitor H89 (**b**) for 30 min, and then treated with metformin and liraglutide for 2 h, followed by exposure to PA for additional 21.5 h. Endothelial function including ROS production (upper panel), NO level (middle panel), and p-eNOS protein level (lower panel) were examined. Data are shown as means ± SD. n = 4. *P < 0.05 (vs. control); ^#^*P* < 0.05 (vs. PA); ^†^*P* < 0.05 (vs. PA + metformin + liraglutide). Met, metformin; Lira, liraglutide; Ex-9, exendin (9–39).

The vascular protective effects of liraglutide could be improved by metformin, as demonstrated in our *in vitro* and *in vivo* studies. However, the mechanisms related to the synergistic protective effects of this combination therapy remains unclear. GLP-1R and its downstream PKA signalling are known as the mediators in most of the GLP-1 actions^15^. We first identified the expression of GLP-1R in HUVECs with mouse anti-GLP-1R monoclonal antibody by immunofluorescence (Supplemental Fig. 2). To further determine whether GLP-1R and PKA signalling were involved in the regulation of metformin functions in HUVECs, the protein levels of GLP-1R and phosphorylated PKA (p-PKA) were examined. As shown in Fig. 3a, PA reduced the protein levels of GLP-1R and p-PKA in HUVECs in a concentration-dependent manner. Notably, metformin dose-dependently abolished the inhibitory effects of PA (0.5 mmol/L) on the protein levels of GLP-1R and p-PKA (Fig. 3b). In line with this finding, we also found a significantly elevated GLP-1R protein level in the aorta tissues of HFD-fed ApoE^−/−^ mice treated with metformin alone or in combination with liraglutide (Supplemental Fig. 3). Moreover, both GLP-1R antagonist exendin (9–39) and PKA inhibitor H89 partially eliminated the combined effects of metformin and liraglutide on endothelial function in HUVECs (Fig. 4a,b). These results suggested that metformin upregulated GLP-1R level and PKA phosphorylation, which might account for its synergistic effects with liraglutide.

**Figure 5:**
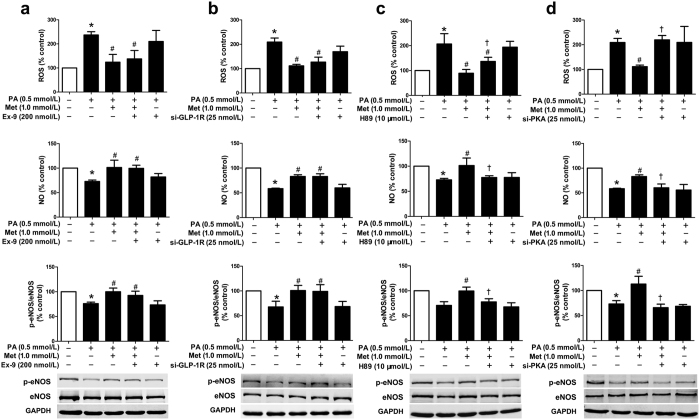
PKA signalling mediated the protective effects of metformin on PA-induced endothelial dysfunction. HUVECs were incubated with exendin (9–39) (**a**) or H89 (**c**) for 30 min, and then treated with metformin for 2 h, followed by exposure to PA for additional 21.5 h. HUV-EC-C, a cell line of HUVECs, was transfected with *GLP-1R* siRNA (si-GLP-1R) (**b**) or *PKA* siRNA (si-PKA) (**d**) for 48 h, and then treated with metformin for 2 h, followed by exposure to PA for additional 22 h. Endothelial function including ROS production (upper panel), NO level (middle panel), and p-eNOS protein level (lower panel) were detected. Data are shown as means ± SD. n = 5. *P < 0.05 (vs. control); ^#^*P* < 0.05 (vs. PA); ^†^*P* < 0.05 (vs. PA + metformin). Met, metformin; Ex-9, exendin (9–39).

To evaluate whether GLP-1R and PKA signalling participated in the protective effect of metformin in HUVECs, siRNAs as well as pharmacological blocking were introduced in our study. In gene knockdown assay, *GLP-1R* siRNA#1 demonstrated a significant decrease in the levels of GLP-1R mRNA and protein (Supplemental Fig. 4a,b), and *PKA* siRNA#1 obviously decreased the protein level of PKA (Supplemental Fig. 4c). Therefore, the siRNA#1 of either GLP-1R or PKA was used in the following experiments. Our data showed that neither exendin (9–39) nor *GLP-1R* siRNA blocked the metformin (1.0 mmol/L)-mediated protective effects in PA-impaired endothelial cells (Fig. 5a,b). By contrast, H89 and *PKA* siRNA attenuated the metformin-mediated protective effects (Fig. 5c,d). Moreover, either exendin (9–39) or *GLP-1R* siRNA could not eliminate the metformin-induced upregulation of p-PKA protein level in PA-treated endothelial cells (Supplemental Fig. 5a,b), suggesting that metformin might upregulate PKA signalling via GLP-1R-independent pathway.

### Metformin restores PA-impaired GLP-1R level and PKA phosphorylation in HUVECs through AMP-activated protein kinase (AMPK) pathway

**Figure 6:**
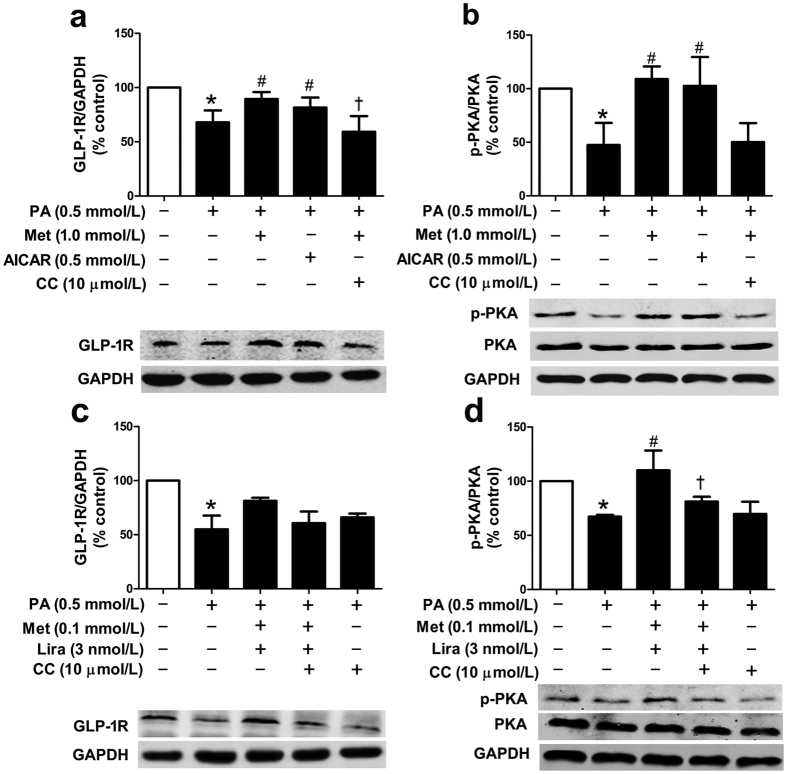
Metformin rescued the impaired GLP-1R level and PKA phosphorylation in PA-treated HUVECs via AMPK pathway. HUVECs were pre-treated with AMPK activator AICAR for 2.5 h, or with AMPK inhibitor compound C for 30 min and then treated with metformin (1.0 mmol/L) alone (**a**,**b**) or metformin (0.1 mmol/L) and liraglutide (3 nmol/L) combination (**c**,**d**) for 2 h, followed by exposure to PA for additional 21.5 h. The protein levels of GLP-1R (**a**,**c**) or p-PKA (**b**,**d**) were normalized to GAPDH or total PKA proteins, respectively. Data are shown as means ± SD. n = 4. *P < 0.05 (vs. control); ^#^*P* < 0.05 (vs. PA); ^†^*P* < 0.05 (vs. PA + metformin or PA + metformin + liraglutide). Met, metformin; Lira, liraglutide; CC, compound C.

It has been reported that metformin exerts its multiple metabolic actions via AMPK pathway^7^. We first examined the effect of either metformin or liraglutide on the protein level of phosphorylated AMPK (p-AMPK) in PA-treated HUVECs. Results showed that metformin could increase p-AMPK protein level in a dose-dependent manner, whereas liraglutide did not have such effect as metformin (Supplemental Fig. 6). Next, AMPK activator AICAR and AMPK inhibitor compound C were used to clarify involvement of AMPK pathway in the metformin-mediated action in PA-treated HUVECs. Our data showed that AICAR (0.5 mmol/L) mimicked, while compound C (10 μmol/L) abolished, the upregulating effects of metformin (1.0 mmol/L) on the protein levels of GLP-1R and p-PKA (Fig. 6a,b). These results suggested that AMPK pathway was involved in the metformin-induced upregulation of GLP-1R level and PKA phosphorylation in PA-impaired HUVECs.

Moreover, compound C was used to investigate whether AMPK pathway was also involved in the combined effect of metformin and liraglutide on the protein levels of GLP-1R and p-PKA. Results showed that combination treatment with lower dose of metformin (0.1 mmol/L) and liraglutide (3 nmol/L) eliminated the inhibitory effects of PA (0.5 mmol/L) on the protein levels of GLP-1R (marginally) and p-PKA (completely). Notably, addition of compound C (10 μmol/L) abolished the combined effect of metformin and liraglutide (Fig. 6c,d). In agreement with this finding, compound C also abolished the synergistic ameliorating effect of combination treatment with lower dose of metformin and liraglutide on the PA-induced reduction of p-eNOS protein level (Supplemental Fig. 7).

## Discussion

Endothelial dysfunction can be considered as a valuable biomarker for predicting atherosclerosis and other cardiovascular lesions^10^. A causal link between free fatty acid and endothelial dysfunction has been established by multiple pathways^16^,^17^,^18^,^19^. PA is one of the fatty acids, which are most commonly found in the western diet. In our *in vitro* study, PA markedly impaired endothelial function as indicated by increased intracellular ROS production, and decreased NO level and eNOS phosphorylation. Likewise, our *in vivo* study demonstrated that Ach-induced endothelium-dependent vasodilation was impaired in HFD-fed ApoE^−/−^ mice.

It has been documented that there are some complementary actions between metformin and GLP-1-based drugs such as GLP-1 analogue and dipeptidyl peptidase 4 inhibitor^20^. Several studies show that metformin can increase plasma active GLP-1 level through enhancing GLP-1 secretion from intestinal L cells^21^ or inhibiting dipeptidyl peptidase 4-mediated GLP-1 degradation^22^,^23^. Combination of metformin and GLP-1-based agents has additive or even synergistic effects on several metabolic parameters^20^. Since either metformin or GLP-1 analogue has vascular protective effects^7^,^24^, we reasonably hypothesized that this combination therapy might also have a synergistic protective effect on endothelial function. Our *in vitro* study found that compared to the single treatment, combination treatment with metformin and liraglutide was more effective to prevent PA-induced endothelial dysfunction, as indicated by inhibiting ROS production, and increasing NO level and eNOS phosphorylation. Furthermore, our *in vivo* study also showed that co-administration of the two drugs in HFD-fed ApoE^−/−^ mice had add-on effects in improving vascular endothelial function and enhancing aortic p-eNOS protein level. Our results are consistent with the findings in human aortic endothelial cells as reported recently. As shown in that report, combination treatment with metformin and liraglutide can ameliorate high glucose-induced oxidative stress via inhibition of PKC-NAD(P)H oxidase pathway^25^.

GLP-1R downregulation can diminish the effects of GLP-1 and its analogues on target cells. It has been shown that GLP-1R level is decreased in some injured cells, such as in human and mouse hepatocytes of individuals with non-alcoholic steatohepatitis^26^,^27^. Moreover, downregulation of GLP-1R level is also found in the glomerular endothelial cells of diabetic mice, which may be due to increased GLP-1R degradation resulted from diabetes-induced PKCβ2 activation^28^. Likewise, our *in vitro* study showed that GLP-1R protein level in HUVECs was significantly decreased after exposure to PA for 24 h. Notably, metformin can directly upregulate GLP-1R expression in pancreatic β cells^29^,^30^,^31^. Similarly, our study revealed that metformin increased GLP-1R protein level in PA-treated HUVECs. We also found that metformin attenuated the PA-induced downregulation of PKA phosphorylation in HUVECs. As is well known, PKA is a downstream signalling of GLP-1R^32^. Thus, we anticipated that metformin could upregulate GLP-1R and PKA signalling to exert its synergistic protective effects with liraglutide in PA-impaired endothelial cells. As we expected, either GLP-1R blockade or PKA inhibition partially eliminated the protective effects of the combined therapy on endothelial function. We also evaluated whether GLP-1R and PKA signalling were involved in the protective effect of metformin in HUVECs. We showed that H89 or *PKA* siRNA blocked the protective effects of metformin on PA-induced endothelial dysfunction, suggesting that metformin alone might upregulate PKA signalling. Although metformin increased GLP-1R level, exendin (9–39) or *GLP-1R* siRNA did not attenuate the protective effects of metformin in PA-impaired HUVECs, suggesting that metformin *per se* could not exert protective effects via GLP-1R, instead, metformin upregulated GLP-1R to enhance the protective effects of liraglutide on endothelial function. Taken together, metformin may upregulate PKA signalling to exert its synergistic protective effects with liraglutide in both GLP-1R-dependent and -independent manners in PA-impaired endothelial cells.

Whether GLP-1R is expressed in endothelial cells remains controversial^28^,^33^,^34^,^35^. Richards and colleagues^33^ generated GLP-1R-Cre mice crossed with fluorescent reporter strains for identification and characterization of live cells expressing GLP-1R. Results showed that GLP-1R was detectable in vasculature, but mainly in smooth muscle. However, it is possible that cells weakly expressing GLP-1R may generate insufficient Cre recombinase to activate the fluorescent reporters. Data from other groups and our recent study have shown that endothelium and cardiac and vascular myocytes express a functional GLP-1R, and participate in the protective cardiovascular effects of GLP-1 analogues^11^,^36^,^37^,^38^. In the present study, GLP-1R expression in human endothelial cells was detected by RT-PCR and western blot analyses, and we demonstrated that GLP-1R at both mRNA and protein levels was detectable, albeit not very strong, in the human endothelial cells. It has been reported that the commercially available antibodies are thought to be nonspecific or inaccurate for GLP-1R protein detection^33^. Recently, Pyke and colleagues^39^ created a mouse anti-GLP-1R monoclonal antibody, which is highly sensitive and specific in the detection of GLP-1R in primate tissues. Therefore, we examined the expression of GLP-1R by using this antibody. Results showed that GLP-1R was expressed in primary HUVECs as detected by both immunofluorescence and western blot analyses. Next, we explored how metformin regulated GLP-1R level and PKA signalling. Pan and colleagues^29^ revealed that AMPK pathway was involved in the metformin-mediated upregulation of GLP-1R mRNA and protein levels in INS-1, a rat β cell line. However, in another study using an INS-1 832/3 β cell line, the metformin-induced upregulation of GLP-1R mRNA expression was mediated via peroxisome proliferator-activated receptor-α rather than AMPK pathway^30^. Moreover, a recent study reported that activation of peroxisome proliferator-activated receptor-β/δ could protect rat pancreatic β cells from PA-induced apoptosis through upregulation of GLP-1R expression^31^. In this study, the PA-induced reduction of GLP-1R level and PKA phosphorylation in HUVECs was reversed by either metformin or AMPK activator AICAR. Importantly, the upregulating effects of metformin on GLP-1R level and PKA phosphorylation were eliminated by adding AMPK inhibitor compound C. These results indicate that AMPK pathway is involved in the regulation of metformin on GLP-1R level and PKA phosphorylation in PA-treated HUVECs. However, how AMPK regulates GLP-1 signalling pathway needs to be further investigated.

AMPK and PKA are two key regulators of cellular metabolism. Interaction between AMPK and PKA has been well documented in several studies^40^,^41^,^42^. AMPK activation increases PKA activity, while PKA independently or in turn inhibits the activity of *Ser*/*Thr*-specific protein phosphatase-2C, thereby indirectly modulating AMPK activity in rat primary vascular smooth muscle cells^41^. In this study, we found that AMPK activation attenuated the PA-induced reduction of PKA phosphorylation in HUVECs. Our findings may shed new light on the signalling network in endothelial cells. In addition, PKA inhibition attenuated the effects of metformin on PA-induced endothelial dysfunction, suggesting that PKA may be involved in the protective effects of metformin on endothelial cells. Nevertheless, further studies are needed to ascertain the crosstalk between AMPK and PKA pathways for the improvement of endothelial function.

**Figure 7:**
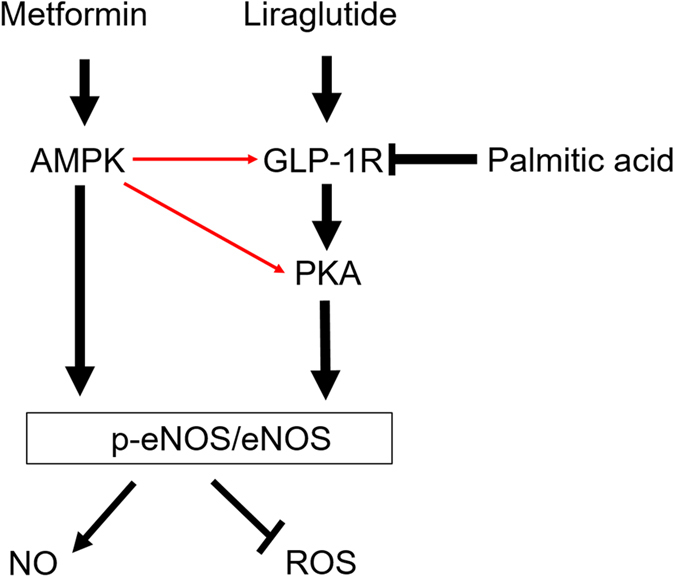
Proposed scheme for the synergistic protective effects of metformin and liraglutide in lipotoxicity-induced endothelial dysfunction. Palmitic acid (PA)-induced endothelial dysfunction is characterized by increased ROS production, decreased NO level and reduced eNOS phosphorylation. Treatment with metformin or liraglutide alone can prevent endothelial cells from the PA-induced dysfunction. Combination treatment with metformin and liraglutide has a synergistic effect on the PA-induced endothelial dysfunction. In addition, metformin upregulates GLP-1R and its downstream PKA signalling, which may account for its synergistic protective effects with liraglutide in the PA-impaired endothelial cells. Metformin may also activate PKA signalling in a GLP-1R-independent manner. The above effects of metformin are mediated via AMPK pathway. The red arrows denote our novel findings in the present study.

In summary, combination treatment with metformin and liraglutide, a GLP-1 analogue, has a synergistic effect against PA-induced endothelial dysfunction. The function of metformin in upregulating GLP-1R level and PKA phosphorylation in endothelial cells may contribute to the synergistic protective effect of this combination therapy, which provides new insights on the interaction between metformin and GLP-1 agents (Fig. 7). It is conceivable that metformin may be able to potentiate or strengthen the vascular protective effects of GLP-1 and its analogues. Our findings provide important information for designing new GLP-1-based therapy strategies in the treatment of type 2 diabetes.

## Materials and Methods

### Reagents

Liraglutide was provided by Novo Nordisk (Bagsvaerd, Denmark). PA, AMPK inhibitor compound C and GLP-1R antagonist exendin (9–39) were purchased from Sigma (St. Louis, MO, USA). AMPK activator AICAR was from Beyotime Biotechnology (Shanghai, China). PKA inhibitor H89 was provided by Cell Signalling Technology (Beverly, MA, USA). Mouse anti-eNOS and anti-phospho-Ser-1177-eNOS (p-eNOS) antibodies were from BD Biosciences (San Joe, CA, USA). Mouse anti-GLP-1R monoclonal antibody was from DSHB (Iowa, IA, USA). Rabbit anti-PKA Cα, anti-phospho-Thr197-PKA C (p-PKA), anti-AMPKα and anti-phospho-Thr-172-AMPKα (p-AMPK) antibodies were obtained from Cell Signalling Technology. Mouse anti-GAPDH antibody from Zhongshan Biotechnology (Beijing, China).

### *In vitro* studies

#### PA preparation

PA of 256 mg was dissolved in 5 mL absolute ethanol and then titrated with 0.1 mol/L sodium hydroxide 5 mL at 70 °C. Finally, 10 mL PA mixture was added with 190 mL 10% BSA at 55 °C to make a complex at the concentrations of 5 mmol/L. The stock solution was filter-sterilized and stored at −20 °C. Control solution containing 5% ethanol and 9.5% BSA was similarly prepared.

#### Cell culture and treatment

HUVECs were collected from healthy donors with written informed consent. The protocol of this study was approved by the Ethical Committee of Peking University Third Hospital. All procedures performed were in accordance with the relevant guidelines and regulations. HUVECs were prepared and cultured as previously reported^43^. All of the experiments were performed using cells at passage 6. A series of PA concentrations (0.125, 0.25 and 0.5 mmol/L) were used for 24 h incubation to induce endothelial dysfunction. In most of our study, exposure of HUVECs to 0.5 mmol/L PA was used as an injured model. Pre-treatment of HUVECs with metformin (0.1, 0.5 and 1.0 mmol/L) or liraglutide (3, 10, 30 and 100 nmol/L) was performed 2 h prior to the PA exposure and in 22 h duration. In the combination experiment, cells were treated with both metformin (0.1 mmol/L) and liraglutide (3 nmol/L). To clarify the involved signalling pathways, cells were incubated with AICAR (0.5 mmol/L), compound C (10 μmol/L), exendin (9–39) (200 nmol/L) or H89 (10 μmol/L) for 30 min before other treatments.

In knockdown experiment, HUV-EC-C, a cell line of HUVECs, was used. This cell line was from Cell Resource Centre of Shanghai Life Science Research Institute (China), and was cultured in DMEM (Invitrogen, Carlsbad, CA, USA) supplemented with 10% FBS.

#### RNA interference

To silence *GLP-1R* and *PKA* gene expression, cells were transfected with siRNAs using lipofectamine RNAi MAX reagent (Invitrogen). For each specific gene knockdown, three forms of siRNAs were included, and an unrelated, scrambled siRNA without any match in human genomic sequence was used as a control. After transfection for 48 h, cells were treated with metformin (1.0 mmol/L) for 2 h, followed by the PA exposure for additional 22 h.

#### Detection of intracellular ROS

ROS was measured using the fluorescent probe 2′,7′-dichlorofluorescin diacetate (DCFH-DA, Sigma), which can be hydrolyzed to DCFH within cells. When the non-fluorescent DCFH is oxidized by ROS, it will be converted to highly fluorescent DCF. Briefly, by the end of treatment, cells were incubated with DCFH-DA (20 μmol/L) for 30 min at 37 °C, and then washed twice. The DCF fluorescent intensity was assayed by a flow cytometer (BD Biosciences). Controls were setup as 100% of the ROS levels.

#### Detection of supernatant NO

NO was determined by nitrite reductase method, using a kit from Beijing 4A Biotech Co., Ltd (China). At the end of treatment, the cell culture supernatants of each group were collected. To set up the assay, tubes with 0.1 mL distilled water, standard solution and the treatment samples were prepared, and then PBS and nitrate reductase were added into each tube. After incubation for 1 h at 37 °C, two working solutions were added for co-incubation for another 10 min. Absorbance at 450 nm in each tube was taken using a microplate reader. The NO level of each group was normalized to its total protein.

#### Western blot

Proteins were separated by 10% (wt/vol) SDS-PAGE and transferred to a nitrocellulose membrane. Membranes were probed with primary antibodies (all at 1:1,000 dilution) overnight at 4 °C, and subsequently incubated with IRDye 800CW-conjugated goat anti-rabbit IgG or goat anti-mouse IgG (both at 1:10,000 dilution; LI-COR Biosciences, Lincoln, NE, USA) for 1 h. Protein bands were visualized with Odyssey infrared imaging system (LI-COR Biosciences).

### *In vivo* studies

#### Animals

The animal experiments were approved by the Animal Care and Use Committee of Peking University and were conducted in accordance with national and international guidelines. Eight-week-old male wild-type and ApoE^−/−^ mice (Vital River Animal Centre, Beijing, China) were housed under specific pathogen-free conditions, with a 12 h light-dark cycle. Experiments were performed after one week’s acclimatization. Wild-type mice were fed with normal diet, while ApoE^−/−^ mice were fed with HFD containing 0.15% cholesterol and 41% fat (MD12015, Medicine Ltd, Yangzhou, Jiangsu, China) for eight weeks. During the final four weeks, the ApoE^−/−^ mice were divided into four groups (6 mice per group), including control (PBS, ip, twice daily), metformin (100 mg/kg per day, via drinking water; and PBS, ip, twice daily), liraglutide (30 μg/kg per day, ip, twice daily) and combination (metformin, 100 mg/kg per day, via drinking water; and liraglutide, 30 μg/kg per day, ip, twice daily). Body weights were measured before treatment and then once a week till sacrificed.

#### Preparation of aortic rings and measurement of vasodilation

After midline laparotomy under pentobarbital sodium anesthesia, aorta was rapidly excised for vascular reactivity measurements as previously reported^44^. After stabilized in Krebs’ buffer for 30 min, aortic rings were depolarized with potassium chloride (60 mmol/L) for evaluation of their maximal contraction. Following two washes, aortic rings were contracted by a stimulation using phenylephrine (10^−6^ mol/L). When the contractile response was stabilized (steady-state phase, 15 min), vasorelaxing responses to an increased concentration of Ach were examined. The resting tension of aortic rings was adjusted to 1.0 *g*. Changes in vascular tension were detected by a pen-writing recorder (ADInstruments, Bella Vista, Australia).

#### HE and immunochemistry staining

Aortic tissues including aortic roots and ascending aorta were collected for preparation of 7 μm thick sections. Tissue sections were firstly identified by HE staining. For immunochemistry staining, the sections were treated with 3% hydrogen peroxide for 10 min to block endogenous peroxidase activity, and incubated in buffered normal horse serum to prevent the non-specific binding of antibodies. The sections were then incubated with antibody against p-eNOS (1:50; Sigma) or GLP-1R (1:10) overnight at 4 °C and subjected to immunohistochemical analysis.

### Statistical analysis

Data are presented as means ± SD. Difference between groups was analysed by one-way ANOVA followed by the post-hoc Tukey-Kramer test. A *P* value < 0.05 was considered as statistically significant. All analyses were performed using SPSS 20.0 for Windows (SPSS Japan Inc., Tokyo, Japan).

## Additional Information

**How to cite this article**: Ke, J. *et al*. Synergistic effects of metformin with liraglutide against endothelial dysfunction through GLP-1 receptor and PKA signalling pathway. *Sci. Rep.* **7**, 41085; doi: 10.1038/srep41085 (2017).

**Publisher's note:** Springer Nature remains neutral with regard to jurisdictional claims in published maps and institutional affiliations.

## Supplementary Material

Supplementary Information

## References

[b1] Woodward M. Use of national data sources in diabetes epidemiology. Lancet Diabetes Endocrinol. 3, 92–93 (2015). 25466720 10.1016/S2213-8587(14)70237-2

[b2] Shah A. D. . Type 2 diabetes and incidence of cardiovascular diseases: a cohort study in 1.9 million people. Lancet Diabetes Endocrinol. 3, 105–113 (2015). 25466521 10.1016/S2213-8587(14)70219-0 PMC4303913

[b3] Hamilton S. J. & Watts G. F. Endothelial dysfunction in diabetes: pathogenesis, significance, and treatment. Rev. Diabet. Stud. 10, 133–156 (2013). 24380089 10.1900/RDS.2013.10.133 PMC4063100

[b4] Viollet B. . Cellular and molecular mechanisms of metformin: an overview. Clin. Sci. (Lond.) 122, 253–270 (2012). 22117616 10.1042/CS20110386 PMC3398862

[b5] Bailey C. J. Metformin: effects on micro and macrovascular complications in type 2 diabetes. Cardiovasc. Drugs Ther. 22, 215–224 (2008). 18288595 10.1007/s10557-008-6092-0

[b6] Holman R. R., Paul S. K., Bethel M. A., Matthews D. R. & Neil H. A. 10-year follow-up of intensive glucose control in type 2 diabetes. N. Engl. J. Med. 359, 1577–1589 (2008). 18784090 10.1056/NEJMoa0806470

[b7] Anfossi G., Russo I., Bonomo K. & Trovati M. The cardiovascular effects of metformin: further reasons to consider an old drug as a cornerstone in the therapy of type 2 diabetes mellitus. Curr. Vasc. Pharmacol. 8, 327–337 (2010). 19485923 10.2174/157016110791112359

[b8] Straughan J. L. Focus on metformin-a major cardiovascular medication. ‘Diabesity-the biggest epidemic in human history’. Cardiovasc. J. Afr. 18, 331–333 (2007). 17985034

[b9] Marso S. P. . Liraglutide and cardiovascular outcomes in type 2 diabetes. N. Engl. J. Med. 375, 311–322 (2016). 27295427 10.1056/NEJMoa1603827 PMC4985288

[b10] Halcox J. P. . Prognostic value of coronary vascular endothelial dysfunction. Circulation 106, 653–658 (2002). 12163423 10.1161/01.cir.0000025404.78001.d8

[b11] Koska J. . Exenatide protects against glucose- and lipid-induced endothelial dysfunction: evidence for direct vasodilation effect of GLP-1 receptor agonists in humans. Diabetes 64, 2624–2635 (2015). 25720388 10.2337/db14-0976 PMC4477348

[b12] Nauck M., Frid A. & Hermansen K. Efficacy and safety comparison of liraglutide, glimepiride, and placebo, all in combination with metformin, in type 2 diabetes: the LEAD (liraglutide effect and action in diabetes)-2 study. Diabetes Care 32, 84–90 (2009). 18931095 10.2337/dc08-1355 PMC2606836

[b13] Nauck M. . Long-term efficacy and safety comparison of liraglutide, glimepiride and placebo, all in combination with metformin in type 2 diabetes: 2-year results from the LEAD-2 study. Diabetes Obes. Metab. 15, 204–212 (2013). 22985213 10.1111/dom.12012

[b14] Forst T. . Addition of liraglutide in patients with Type 2 diabetes well controlled on metformin monotherapy improves several markers of vascular function. Diabet. Med. 29, 1115–1118 (2012). 22288732 10.1111/j.1464-5491.2012.03589.x

[b15] Arakawa M. . Inhibition of monocyte adhesion to endothelial cells and attenuation of atherosclerotic lesion by a glucagon-like peptide-1 receptor agonist, exendin-4. Diabetes 59, 1030–1037 (2010). 20068138 10.2337/db09-1694 PMC2844811

[b16] Wang X. L. . Free fatty acids inhibit insulin signaling-stimulated endothelial nitric oxide synthase activation through upregulating PTEN or inhibiting Akt kinase. Diabetes 55, 2301–2310 (2006). 16873694 10.2337/db05-1574

[b17] Steinberg H. O. & Baron A. D. Vascular function, insulin resistance and fatty acids. Diabetologia 45, 623–634 (2002). 12107742 10.1007/s00125-002-0800-2

[b18] Mehra V. C. . Ceramide-activated phosphatase mediates fatty acid-induced endothelial VEGF resistance and impaired angiogenesis. Am. J. Pathol. 184, 1562–1576 (2014). 24606881 10.1016/j.ajpath.2014.01.009 PMC4005977

[b19] Kajikawa M. . Ratio of serum levels of AGEs to soluble form of RAGE is a predictor of endothelial function. Diabetes Care 38, 119–125 (2015). 25336748 10.2337/dc14-1435

[b20] Liu Y. & Hong T. Combination therapy of dipeptidyl peptidase-4 inhibitors and metformin in type 2 diabetes: rationale and evidence. Diabetes Obes. Metab. 16, 111–117 (2014). 10.1111/dom.12128 23668534

[b21] Mulherin A. J. . Mechanisms underlying metformin-induced secretion of glucagon-like peptide-1 from the intestinal L cell. Endocrinology 152, 4610–4619 (2011). 21971158 10.1210/en.2011-1485

[b22] Lindsay J. R. . Inhibition of dipeptidyl peptidase IV activity by oral metformin in type 2 diabetes. Diabet. Med. 22, 654–657 (2005). 15842525 10.1111/j.1464-5491.2005.01461.x

[b23] Fadini G. P., Albiero M., Menegazzo L., de Kreutzenberg S. V. & Avogaro A. The increased dipeptidyl peptidase-4 activity is not counteracted by optimized glucose control in type 2 diabetes, but is lower in metformin-treated patients. Diabetes Obes. Metab. 14, 518–522 (2012). 22171692 10.1111/j.1463-1326.2011.01550.x

[b24] Anagnostis P. . Glucagon-like peptide-1-based therapies and cardiovascular disease: looking beyond glycaemic control. Diabetes Obes. Metab. 13, 302–312 (2011). 21205117 10.1111/j.1463-1326.2010.01345.x

[b25] Batchuluun B. . Metformin and liraglutide ameliorate high glucose-induced oxidative stress via inhibition of PKC-NAD(P)H oxidase pathway in human aortic endothelial cells. Atherosclerosis 232, 156–164 (2014). 24401231 10.1016/j.atherosclerosis.2013.10.025

[b26] Svegliati-Baroni G. . Glucagon-like peptide-1 receptor activation stimulates hepatic lipid oxidation and restores hepatic signalling alteration induced by a high-fat diet in nonalcoholic steatohepatitis. Liver Int. 31, 1285–1297 (2011). 21745271 10.1111/j.1478-3231.2011.02462.x

[b27] Gupta N. A. . Glucagon-like peptide-1 receptor is present on human hepatocytes and has a direct role in decreasing hepatic steatosis *in vitro* by modulating elements of the insulin signaling pathway. Hepatology 51, 1584–1592 (2010). 20225248 10.1002/hep.23569 PMC2862093

[b28] Mima A. . Protective effects of GLP-1 on glomerular endothelium and its inhibition by PKCβ activation in diabetes. Diabetes 61, 2967–2979 (2012). 22826029 10.2337/db11-1824 PMC3478518

[b29] Pan Q. R. . Glucose, metformin, and AICAR regulate the expression of G protein-coupled receptor members in INS-1 beta cell. Horm. Metab. Res. 41, 799–804 (2009). 19672815 10.1055/s-0029-1234043

[b30] Maida A., Lamont B. J., Cao X. & Drucker D. J. Metformin regulates the incretin receptor axis via a pathway dependent on peroxisome proliferator-activated receptor-alpha in mice. Diabetologia 54, 339–349 (2011). 20972533 10.1007/s00125-010-1937-z

[b31] Yang Y. . Activation of PPARβ/δ protects pancreatic β cells from palmitate-induced apoptosis by upregulating the expression of GLP-1 receptor. Cell Signal. 26, 268–278 (2014). 24269940 10.1016/j.cellsig.2013.11.019

[b32] Goodwill A. G. . Cardiovascular and hemodynamic effects of glucagon-like peptide-1. Rev. Endocr. Metab. Disord. 15, 209–217 (2014). 24881624 10.1007/s11154-014-9290-z PMC4119853

[b33] Richards P. . Identification and characterization of GLP-1 receptor-expressing cells using a new transgenic mouse model. Diabetes 63, 1224–1233 (2014). 24296712 10.2337/db13-1440 PMC4092212

[b34] Ussher J. R. & Drucker D. J. Cardiovascular actions of incretin-based therapies. Circ. Res. 114, 1788–1803 (2014). 24855202 10.1161/CIRCRESAHA.114.301958

[b35] Fujita H. . The protective roles of GLP-1R signaling in diabetic nephropathy: possible mechanism and therapeutic potential. Kidney Int. 85, 579–589 (2014). 24152968 10.1038/ki.2013.427

[b36] Wei R. . Exenatide exerts direct protective effects on endothelial cells through the AMPK/Akt/eNOS pathway in a GLP-1 receptor-dependent manner. Am. J. Physiol. Endocrinol. Metab. 310, E947–957 (2016). 27072494 10.1152/ajpendo.00400.2015

[b37] Chen W. R. . Effects of liraglutide on left ventricular function in patients with ST-segment elevation myocardial infarction undergoing primary percutaneous coronary intervention. Am. Heart J. 170, 845–854 (2015). 26542491 10.1016/j.ahj.2015.07.014

[b38] Lonborg J. . Impact of acute hyperglycemia on myocardial infarct size, area at risk, and salvage in patients with STEMI and the association with exenatide treatment: results from a randomized study. Diabetes 63, 2474–2485 (2014). 24584550 10.2337/db13-1849

[b39] Pyke C. . GLP-1 receptor localization in monkey and human tissue: novel distribution revealed with extensively validated monoclonal antibody. Endocrinology 155, 1280–1290 (2014). 24467746 10.1210/en.2013-1934

[b40] Yin W., Mu J. & Birnbaum M. J. Role of AMP-activated protein kinase in cyclic AMP-dependent lipolysis In 3T3-L1 adipocytes. J. Biol. Chem. 278, 43074–43080 (2003). 12941946 10.1074/jbc.M308484200

[b41] Stone J. D., Narine A. & Tulis D. A. Inhibition of vascular smooth muscle growth via signaling crosstalk between AMP-activated protein kinase and cAMP-dependent protein kinase. Front. Physiol. 3, 409 (2012). 23112775 10.3389/fphys.2012.00409 PMC3482697

[b42] Hurley R. L. . Regulation of AMP-activated protein kinase by multisite phosphorylation in response to agents that elevate cellular cAMP. J. Biol. Chem. 281, 36662–36672 (2006). 17023420 10.1074/jbc.M606676200

[b43] Lantoine F., Iouzalen L., Devynck M. A., Millanvoye-Van Brussel E. & David-Dufilho M. Nitric oxide production in human endothelial cells stimulated by histamine requires Ca^2+^ influx. Biochem. J. 330 (Pt 2), 695–699 (1998). 9480877 10.1042/bj3300695 PMC1219192

[b44] Chinen I. . Vascular lipotoxicity: endothelial dysfunction via fatty-acid-induced reactive oxygen species overproduction in obese Zucker diabetic fatty rats. Endocrinology 148, 160–165 (2007). 17023526 10.1210/en.2006-1132

