# Supplementary material for: Synergistic effects of metformin with liraglutide against endothelial dysfunction through GLP-1 receptor and PKA signalling pathway
Source: Sci Rep. 2017 Feb 1;7:41085. doi: 10.1038/srep41085 (PMC5286534; doi:10.1038/srep41085)
Supplement: Supplementary Information [file srep41085-s1.pdf]

Supplementary Information

Synergistic effects of metformin with liraglutide against endothelial dysfunction through GLP-1 receptor and PKA signalling pathway

Jing Ke, Ye Liu, Jin Yang, Ran Lu, Qing Tian, Wenfang Hou, Guang Wang, Rui Wei, Tianpei Hong

Supplementary Methods

Measurement of intracellular ROS by confocal microscope

Cells were incubated for 24 h under different treatment conditions, and were subsequently incubated with DCFH-DA (20 μmol/L) in serum-free medium for 30 min at 37°C, followed by two washes with PBS. Fluorescent intensity was observed and assayed by a laser confocal microscope (LSM 510 META, Carl Zeiss, Jena, Germany). Controls were set up as 100% of the intracellular ROS levels.

Detection of intracellular NO by flow cytometry

Intracellular NO levels were measured using a NO-sensitive fluorescence probe 3-amino, 4-aminomethyl-2', 7'-difluorescein, diacetate (DAF-FM DA) (Beyotime Institute of Biotechnology, Shanghai, China) according to the manufacturer’s instructions. Cells were incubated in 60-mm plates for 24 h under different treatment conditions, and were subsequently incubated with DAF-FM DA (5 μmol/L) in serum-free medium for 30 min at 37°C, and then washed with PBS and gently trypsinized. Fluorescent intensity was assayed by a flow cytometer (BD Biosciences, San Joe, CA, USA) at an emission wavelength of 515 nm and an excitation wavelength of 495 nm. Controls were set up as 100% of the intracellular NO levels.

Immunofluorescence

The paraformaldehyde-prefixed HUVECs were immunostained with mouse anti-GLP-1R monoclonal antibody (7 μg/ml, DSHB) overnight at 4°C. After three washes, cells were then incubated with Alexa Fluor 594-conjugated goat anti-mouse IgG (1:800; Jackson ImmunoResearch, West Grove, PA, USA) for 30 min at room temperature, followed by 4’6’-diamino-2-phenylindole (DAPI, 1 μg/ml;

Sigma) staining after washing. Images were captured under a fluorescent microscope (Leica, Germany). Negative controls were performed by using isotypic sera to replace the primary antibodies.

RT-PCR

RNA of HUV-EC-C, a cell line of HUVECs, was extracted with Trizol (Invitrogen, Carlsbad, CA) and reverse-transcribed to cDNA by using a First Strand cDNA synthesis Kit (Fermentas, Burlington, ON, Canada). The cDNA was amplified by PCR using Taq Plus PCR Master Mix (Qiagen, Duesseldorf, Germany). The primer sequences specific for GLP-1R (480bp) were: forward primer 5´-TCAAGGTCAACGGCTTATTAG-3´and reverse primer 5´-TAACGTGTCCCTAGATGAACC-3´. The primer sequences specific for GAPDH (289 bp) were: forward primer 5´-ACAGTCAGCCGCATCTTCTT-3´ and reverse primer 5´-CTGGAAGATGGTGATGGGAT-3´.

Supplementary Figures and Legends

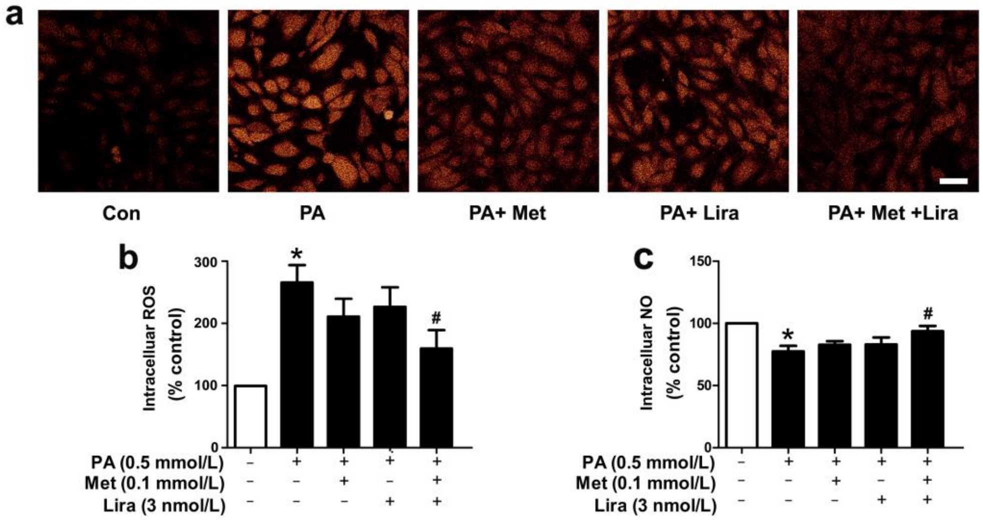

Supplemental Figure 1. Synergistic effects of metformin and liraglutide on PA-induced upregulation of ROS production and reduction of NO generation in primary HUVECs. Cells were incubated for 24 h with metformin (0.1 mmol/L) and/or liraglutide (3 nmol/L) in the presence of a concomitant exposure to PA (0.5 mmol/L). Representative photographs (**a**) and quantification (**b**) of ROS fluorescence intensity as detected by a laser confocal microscope. (**c**) Intracellular NO levels measured by a flow cytometer. Data are shown as means ± SD. n=4. $^{*}P$<0.05 (vs. control); $^{\#}P$<0.05 (vs. PA). Con, control; PA: palmitic acid; Met, metformin; Lira, liraglutide. Scale bars: 100 μm

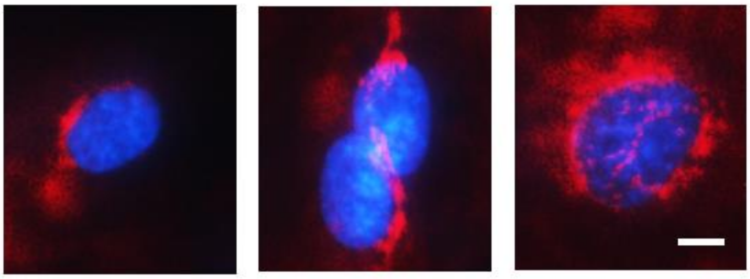

Supplemental Figure 2. Location and expression of GLP-1R in primary HUVECs. Immunofluorescent staining of GLP-1R (red) and nucleus (blue). Scale bars: 10 μm.

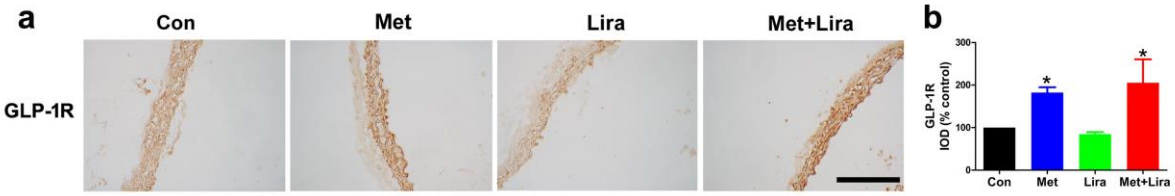

Supplemental Figure 3. Effects of metformin and/or liraglutide on GLP-1R level in aortic tissues of ApoE$^{-/-}$ mice. (**a**) Representative images of GLP-1R immunostaining; (**b**) Quantitation of immunostaining in the vascular tissue. Data are shown as means ± SD. n=6. Con (control), Met (metformin) and Lira (liraglutide) denote HFD-fed ApoE$^{-/-}$ mice without or with the corresponding treatment. $^{*}P$<0.05 (vs. control). Scale bars: 100 μm.

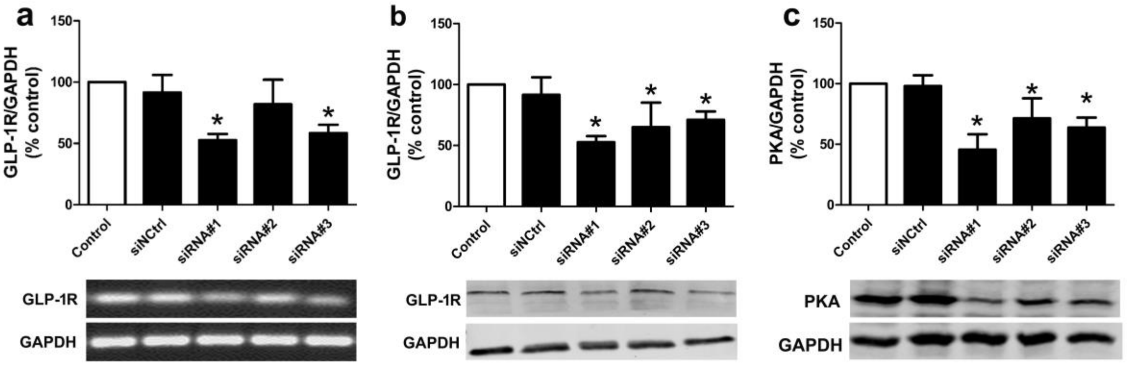

Supplemental Figure 4. Identification of the effects of siRNA in HUV-EC-C, a cell line of HUVECs. Cells were transfected for 48 h with different forms of *GLP-1R* siRNA (si-GLP-1R) or a negative siRNA (siNCtrl), or with different forms of *PKA* siRNA (si-PKA) or the corresponding siNCtrl, respectively. (**a**) GLP-1R mRNA was determined by RT-PCR. The protein levels of GLP-1R (**b**) or PKA (**c**) were analysed by western blot. The levels of GLP-1R and PKA were normalized to GAPDH. Data are shown as means ± SD. n=4. $^{*}P<0.05$ (vs. control).

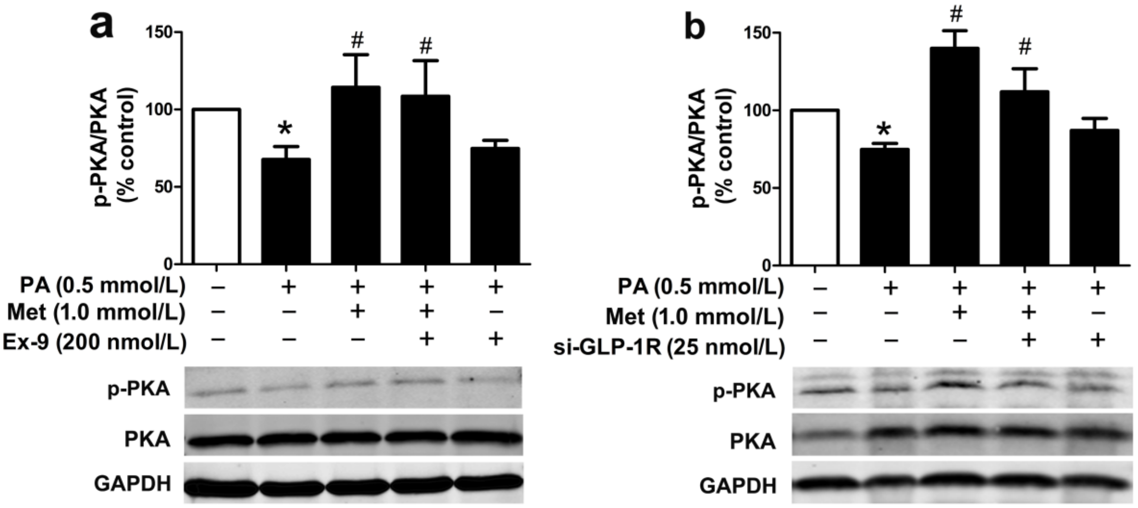

Supplemental Figure 5. Effects of GLP-1R blockade on the metformin-induced upregulation of PKA phosphorylation in PA-treated endothelial cells. (**a**) Primary HUVECs were incubated with the GLP-1R antagonist exendin (9-39) for 30 min, and then treated with metformin (1.0 mmol/L) for 2 h,

followed by exposure to PA (0.5 mmol/L) for additional 21.5 h. (**b**) HUV-EC-C, a cell line of HUVECs, was transfected with a *GLP-1R* siRNA (si-GLP-1R) for 48 h, and then treated with metformin (1.0 mmol/L) for 2 h, followed by exposure to PA (0.5 mmol/L) for additional 22 h. The levels of phosphorylated PKA (p-PKA) were normalized to total PKA proteins. Data are shown as means ± SD. n=4. $^{*}P<0.05$ (vs. control); $^{\#}P<0.05$ (vs. PA). PA, palmitic acid; Met, metformin; Ex-9, exendin (9-39).

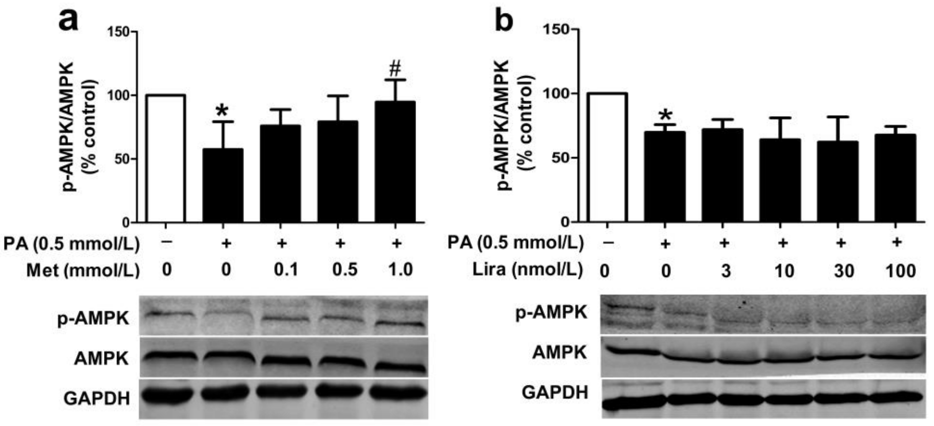

Supplemental Figure 6. Effect of either metformin or liraglutide on AMPK phosphorylation in PA-treated HUVECs. Cells were incubated for 24 h with different concentrations of metformin (**a**) or liraglutide (**b**) in the presence of a concomitant exposure to PA (0.5 mmol/L). The levels of phosphorylated AMPK (p-AMPK) were normalized to total AMPK protein, as detected by western blot. Data are shown as means ± SD. n=3. $^{*}P<0.05$ (vs. control); $^{\#}P<0.05$ (vs. PA). PA, palmitic acid; Met, metformin; Lira, liraglutide.

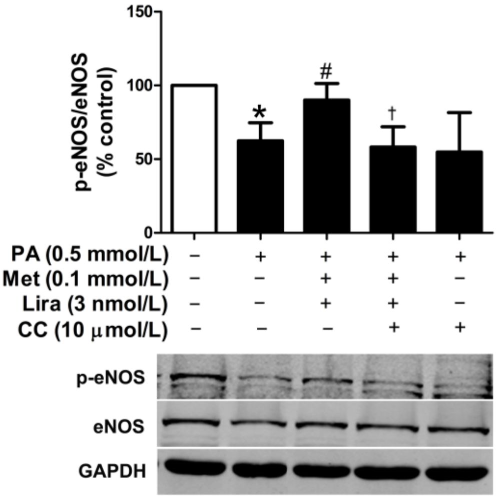

Supplemental Figure 7. AMPK inhibitor compound C abolished the combined effect of metformin and liraglutide on p-eNOS protein level in PA-treated HUVECs. Cells were pre-incubated with compound C (10 μmol/L) for 30 min and then treated for 23.5 h with metformin (0.1 mmol/L) and liraglutide (3 nmol/L) combination in the presence of a concomitant exposure to PA (0.5 mmol/L). Data are shown as means ± SD. n=3. $^{*}P$<0.05 (vs. control); $^{\#}P$<0.05 (vs. PA); $^{\dagger}P$<0.05 (vs. PA + metformin + liraglutide). PA: palmitic acid; Met, metformin; Lira, liraglutide; CC, compound C.
